# Determinants of Restoration of CD4 and CD8 Cell Counts and Their Ratio in HIV-1–Positive Individuals With Sustained Virological Suppression on Antiretroviral Therapy

**DOI:** 10.1097/QAI.0000000000001913

**Published:** 2018-12-03

**Authors:** Luuk Gras, Margaret May, Lars Peter Ryder, Adam Trickey, Marie Helleberg, Niels Obel, Rodolphe Thiebaut, Jodie Guest, John Gill, Heidi Crane, Viviane Dias Lima, Antonella d'Arminio Monforte, Timothy R. Sterling, Jose Miro, Santiago Moreno, Christoph Stephan, Colette Smith, Janet Tate, Leah Shepherd, Mike Saag, Armin Rieger, Daniel Gillor, Matthias Cavassini, Marta Montero, Suzanne M. Ingle, Peter Reiss, Dominique Costagliola, Ferdinand W.N.M. Wit, Jonathan Sterne, Frank de Wolf, Ronald Geskus

**Affiliations:** 1 Stichting HIV Monitoring, Amsterdam, the Netherlands;; 2 Bristol Medical School, University of Bristol, Bristol, United Kingdom;; 3 Tissue Typing Laboratory, Department of Clinical Immunology, Copenhagen University Hospital Rigshospitalet, Copenhagen, Denmark;; 4 Department of Infectious Diseases, Centre of Excellence for Health, Immunity and Infections, Copenhagen University Hospital, Rigshospitalet, Copenhagen, Denmark;; 5 Department of Infectious Diseases, Copenhagen University Hospital, Copenhagen, Denmark;; 6 INSERM, U1219 Bordeaux Population Health Research Centre, Univ. Bordeaux, INRIA SISTM, Bordeaux, France;; 7 School of Public Health and Emory School of Medicine, Atlanta, GA;; 8 Division of Infectious Diseases, University of Calgary, Calgary, Alberta, Canada;; 9 Center for AIDS Research, University of Washington, Seattle, WA;; 10 British Columbia Centre for Excellence in HIV/AIDS, St. Paul's Hospital, Vancouver, British Columbia, Canada;; 11 Division of AIDS, Department of Medicine, Faculty of Medicine, University of British Columbia, Vancouver, British Columbia, Canada;; 12 Clinic of Infectious Diseases and Tropical Medicine, San Paolo Hospital, University of Milan, Milan, Italy;; 13 Vanderbilt University School of Medicine, Nashville, TN;; 14 Infectious Disease Service, Hospital Clínic-IDIBAPS, University of Barcelona, Barcelona, Spain;; 15 Hospital Ramón y Cajal, Madrid, Spain;; 16 Department of Infectious Diseases, University Hospital Frankfurt, Goethe-University, Frankfurt am Main, Germany;; 17 Institute of Global Health, UCL, London, United Kingdom;; 18 Department of Internal Medicine, Yale University School of Medicine, New Haven, CT;; 19 Division of Infectious Diseases, University of Alabama at Birmingham, Birmingham, AL;; 20 University of Vienna, Vienna, Austria;; 21 Universität zu Köln, Cologne, Germany;; 22 Service of Infectious Diseases, Lausanne University Hospital and University of Lausanne, Lausanne, Switzerland;; 23 La Fe Hospital, Valencia, Spain;; 24 Department of Global Health, Academic Medical Center of the University of Amsterdam, Amsterdam, the Netherlands;; 25 Sorbonne Universités UPMC Université Paris 06, INSERM, Institut Pierre Louis d'épidémiologie et de Santé Publique (UMRS 1136), Paris, France;; 26 Amsterdam Institute for Global Health and Development, Amsterdam, the Netherlands;; 27 Department of Infectious Disease Epidemiology, School of Public Health, Imperial College London, London, United Kingdom;; 28 Academic Medical Center of the University of Amsterdam, Amsterdam, the Netherlands;; 29 Public Health Service, Amsterdam, the Netherlands;; 30 Nuffield Department of Medicine, University of Oxford, Oxford, United Kingdom; and; 31 Oxford University Clinical Research Unit, Ho Chi Minh City, Vietnam.

**Keywords:** CD4 cell count, CD8 cell count, CD4:CD8 ratio, antiretroviral therapy, HIV, age

## Abstract

**Background:**

An increasing number of HIV-positive individuals now start antiretroviral therapy (ART) with high CD4 cell counts. We investigated whether this makes restoration of CD4 and CD8 cell counts and the CD4:CD8 ratio during virologically suppressive ART to median levels seen in HIV-uninfected individuals more likely and whether restoration depends on gender, age, and other individual characteristics.

**Methods:**

We determined median and quartile reference values for CD4 and CD8 cell counts and their ratio using cross-sectional data from 2309 HIV-negative individuals. We used longitudinal measurements of 60,997 HIV-positive individuals from the Antiretroviral Therapy Cohort Collaboration in linear mixed-effects models.

**Results:**

When baseline CD4 cell counts were higher, higher long-term CD4 cell counts and CD4:CD8 ratios were reached. Highest long-term CD4 cell counts were observed in middle-aged individuals. During the first 2 years, median CD8 cell counts converged toward median reference values. However, changes were small thereafter and long-term CD8 cell count levels were higher than median reference values. Median 8-year CD8 cell counts were higher when ART was started with <250 CD4 cells/mm^3^. Median CD4:CD8 trajectories did not reach median reference values, even when ART was started at 500 cells/mm^3^.

**Discussion:**

Starting ART with a CD4 cell count of ≥500 cells/mm^3^ makes reaching median reference CD4 cell counts more likely. However, median CD4:CD8 ratio trajectories remained below the median levels of HIV-negative individuals because of persisting high CD8 cell counts. To what extent these subnormal immunological responses affect specific clinical endpoints requires further investigation.

## Abstract 2

Supplemental Digital Content is Available in the Text.

## INTRODUCTION

Since 2012, US Guidelines have recommended offering antiretroviral therapy (ART) to all individuals diagnosed with HIV, regardless of their CD4 cell count.^1^ As a result, an increasing number of HIV-1–positive individuals start ART at high CD4 cell counts. Furthermore, of those starting ART, a considerable proportion does so at a relatively old age. For example, in the Netherlands in 2015, 37% of those starting ART did so with a CD4 count of ≥500 cells/mm^3^ and 23% of individuals newly diagnosed with HIV were aged 50 years or older.^2^ Generally, the increase in CD4 cell count during virologically suppressive ART is less in older individuals.^3–9^ This diminished recovery of CD4 cell count among older individuals has been attributed to lower thymic function.^10,11^

Lower CD4 counts with older age are also seen in healthy European HIV-negative populations, although the decrease seems to occur mainly at a very advanced age.^12–16^ CD4 cell counts have also been reported to differ according to smoking status,^17^ gender,^13^ the time of day of sampling,^18^ season,^19^ and region of origin.^20,21^

Although CD4 cell count is considered the key prognostic factor for AIDS morbidity and mortality, some evidence suggests that the CD4:CD8 ratio also independently predicts time to death and non–AIDS-defining endpoints.^22–24^ In the general population, a CD4:CD8 ratio <1.0 is associated with mortality in very elderly people.^25^ In HIV-positive individuals, the ratio is decreased and low ratios are associated with pathological changes in the immune system such as immune activation, exhaustion, senescence, and memory abnormalities.^26–28^ The ratio increases rapidly during the first few years on ART and keeps increasing up to 15 years after starting ART, albeit slowly,^29^ and the ratio does not reach levels higher than 1.0 in two-thirds of individuals despite long-term viral suppression.^30,31^

We studied whether an early start, at high CD4 cell counts followed by long-term virologically suppressive ART, makes restoration to levels of CD4 and CD8 cell counts and the CD4:CD8 ratio seen in HIV-negative individuals more likely. We also investigated the effect of age and other factors on these immunological changes.

## METHODS

### HIV-Negative Study Participants

To obtain reference values, we used 2309 cross-sectional CD4 and CD8 cell counts and CD4:CD8 ratios obtained from HIV-negative individuals recruited from the background population to the Danish HIV cohort (either healthy staff or blood and stem-cell donors) and HIV-negative individuals from the Dutch AGE_h_IV cohort (recruited either at the STI clinic of the Amsterdam Public Health Service or the existing Amsterdam Cohort Studies on HIV/AIDS). CD4 and CD8 cell counts and CD4:CD8 ratios were used as dependent variables in 3 linear regression models including age and gender and their interaction as independent variables. We used the 25th, 50th, and 75th prediction percentiles as the lower, median, and upper reference values, respectively, in graphs to put the immunological restoration during virologically suppressive ART in HIV-positive individuals into context (see Text File SDC 1, Supplemental Digital Content, http://links.lww.com/QAI/B244 for further details on the selection and analysis of CD4 and CD8 cell counts and the CD4:CD8 ratio in HIV-negative individuals).

### HIV-Positive Study Participants

We used data from the Antiretroviral Therapy Cohort Collaboration (ART-CC; http://www.art-cohort-collaboration.org), an international collaboration of 21 cohort studies from Europe and North America that was established in 2000 to examine the prognosis of HIV-1–positive, treatment-naive individuals initiating ART, a combination of at least 3 antiretroviral drugs.^32^ Participation of cohorts has been approved by their ethics committees or institutional review boards according to local regulations (see Text File SDC 2, Supplemental Digital Content, http://links.lww.com/QAI/B244 for a list of participating cohorts). We only included individuals who were 18 years of age or older and had a CD4 cell count and viral load measured at the start of ART. All included individuals had a decrease in HIV RNA viral load to below 400 copies per milliliter within 9 months from start of ART. In sensitivity analyses, we changed the time limit to 6 months and cutoff to 50 copies per milliliter.

### Outcome

We modeled longitudinal CD4 and CD8 cell counts and CD4:CD8 ratios after the start of ART. We excluded follow-up after an ART interruption longer than 2 weeks and after the first of 2 consecutive plasma viral load measurements ≥400 copies per milliliter. In sensitivity analyses, we only included measurements until an ART interruption longer than 1 week or until the first plasma viral load measurement ≥400 copies per milliliter. Models including CD8 cell counts or CD4:CD8 ratios only included participants from the 14 cohorts that had collected data on these variables.

### Statistical Methods

Trends in CD4 and CD8 cell counts and their ratio were modeled using linear mixed-effects models (lme4 package^33^ in R version 3.0.3^34^). CD4 cell counts were found to best comply with normality assumptions when square root transformed, CD8 cell counts when log transformed, and the CD4:CD8 ratio when fifth root transformed. The trends over time since start of ART were modeled using restricted cubic splines with knots at 0, 0.1, 0.25, 0.5, 3, and 7.5 years. We used a random intercept and 2 random slopes (one slope between 0 and 6 months and one slope from 6 months onward, with an unstructured covariance matrix) per individual as well as a random intercept for cohort.

All models included gender, region of birth (Europe/North America, Caribbean/South America, sub-Saharan Africa, and other regions), transmission risk group [men who have sex with men (MSM), injecting drug use (IDU), heterosexual, other, and unknown], age, CD4 cell count, and HIV RNA at the start of ART (measurement closest to the start of ART in the period 90 days before to 6 days after starting ART). CD4 cell count trends were also allowed to vary according to period of starting ART (2001–2003, 2004–2006, 2007–2009, and 2010–2012).

Because data on smoking status, CD8 cell count, and hepatitis C virus (HCV) and cytomegalovirus (CMV) coinfection were not collected in all cohorts, we only used data from cohorts with at least 85% complete data on smoking status, CD8 cell count, and HCV coinfection. For CMV coinfection, we used data from the 5 cohorts with available data on CMV. Therefore, these variables were not evaluated together in one model but in separate models (For more detailed information on interaction terms and continuous covariable modeling, see Text File SDC 3, Supplemental Digital Content, http://links.lww.com/QAI/B244).

To help interpret, the fitted values were backtransformed to their original scale, where they can be considered as median values. They are graphically displayed for selected values of age and CD4 and CD8 cell counts at the start of ART.

## RESULTS

### HIV-Negative Population

Median CD4 and CD8 cell count in HIV-negative participants decreased with older age, whereas the median CD4:CD8 ratio was higher with older age (see Figures A–C SDC 4, Supplemental Digital Content, http://links.lww.com/QAI/B244). For a 37-year-old man, the median CD4 cell count was 830 cells/mm^3^ and 1005 CD4 cells/mm^3^ for a woman. These values for the CD8 cell count and the CD4:CD8 ratio were 499 cells/mm^3^ and 1.69 for men and 519 CD8 cells/mm^3^ and 1.98 for women, respectively. Modeling age using splines (see Figures SDC 5–7, Supplemental Digital Content, http://links.lww.com/QAI/B244) gave a better data fit than modeling age linearly, but because the resulting trajectories were not consistently increasing or decreasing with higher age, we chose to model age linearly.

### HIV-Positive Population

The majority of 60,997 included HIV-positive individuals was men (75%) and born in Europe/North America (73%), as shown in Table 1. Forty percent were in the MSM transmission risk group. Median age was 39 years [interquartile range (IQR) 32–46]. Median CD4 cell count was 246 cells/mm^3^ (IQR 130–350). The median CD8 cell count in the subset of 37,305 individuals included in the analysis of CD8 cell count and CD4:CD8 ratio was 830 cells/mm^3^ and the median CD4:CD8 ratio was 0.21 (Table 2). Median CD8 cell count and CD4:CD8 ratio were both lower when ART was started at lower CD4 cell count.

**TABLE 1.**
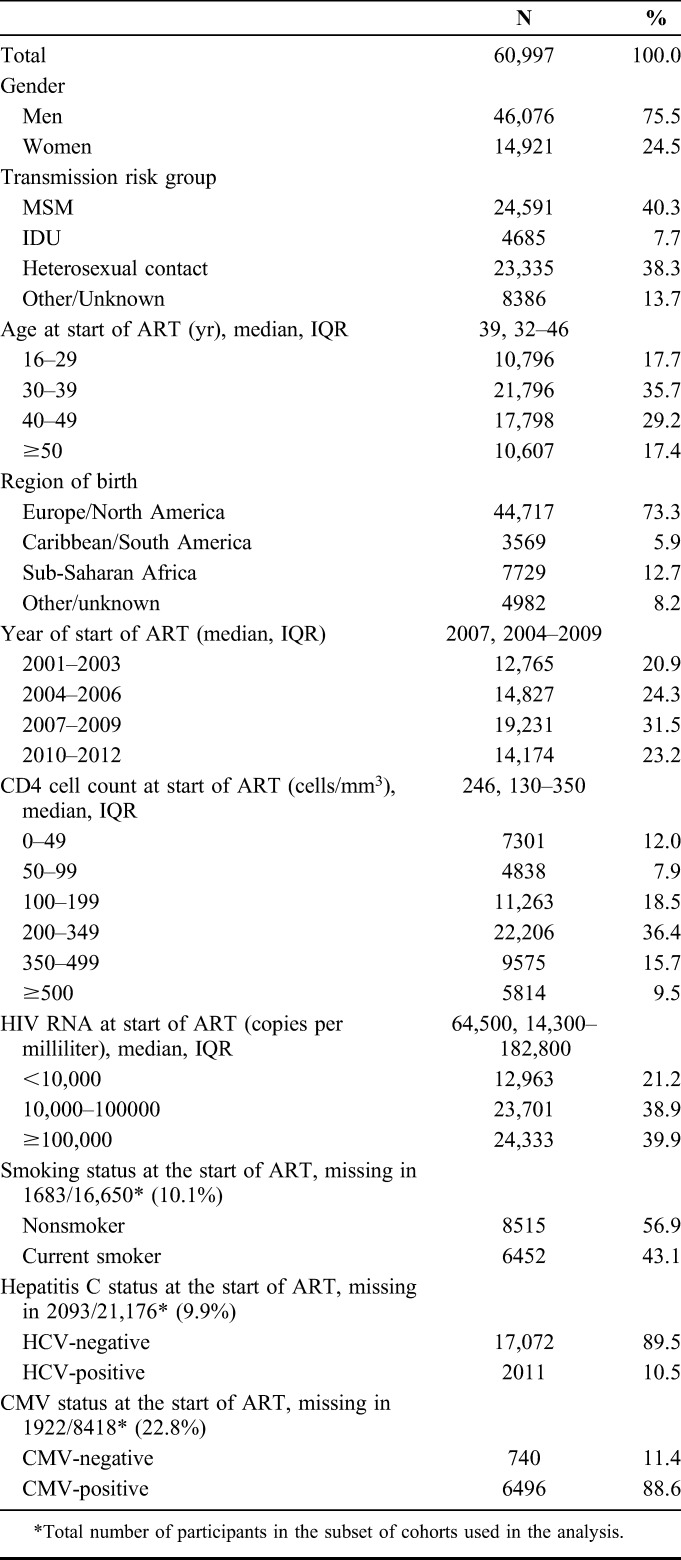
Demographical and Clinical Characteristics at the Start of ART

**TABLE 2.**
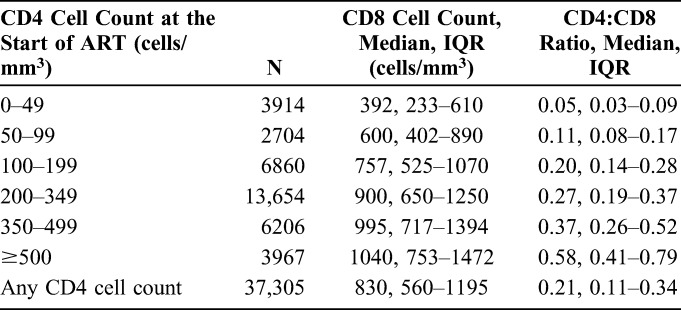
Median (IQR) CD8 Cell Count and CD4:CD8 Ratio at the Start of ART for Different CD4 Categories

### CD4 Cell Count Trajectories

We used 599,445 CD4 cell count measurements. The number of individuals with measurements after 2, 4, 6, and 8 years of virologically suppressive ART was 29,791 (49%), 16,679 (27%), 8836 (14%), and 4209 (7%), respectively. The median observed CD4 cell count at 8 years for those starting with a CD4 count of 0–49 (n = 728), 50–99 (n = 480), 100–199 (n = 1025), 200–349 (n = 1359), 350–499 (n = 377), and ≥500 cells/mm^3^ (n = 240) was 485, 507, 570, 667, 793, and 923 cells/mm^3^, respectively.

**FIGURE 1.**
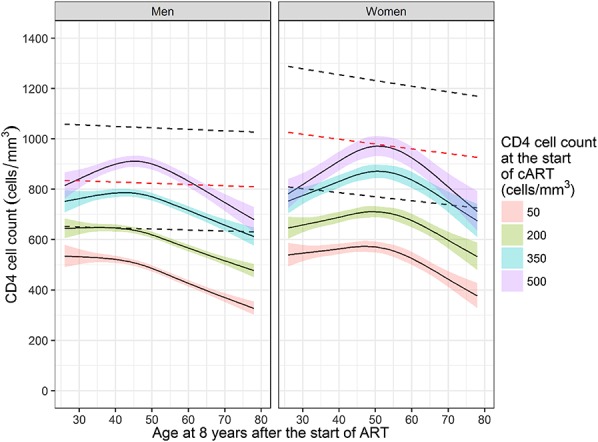
Median CD4 cell count at 8 years of virologically suppressive ART (95% confidence intervals in color) in men and women by age at 8 years after the start of ART. Trends are shown for specific baseline CD4 cell counts of 100, 200, 350, and 500 cells/mm^3^. Curves are for an average reference heterosexual individual born in Western Europe/North America starting ART between 2004 and 2006 with a plasma viral load of 4.81 log_10_ copies per milliliter and random intercept and slopes equal to zero. Dashed lines show the lower, normal and upper reference CD4 cell counts by age as estimated in HIV-negative men and women.

Higher CD4 cell count at the start of ART was associated with higher median 8-year counts (Fig. 1). Only when ART was started with a CD4 count of 500 cells/mm^3^, the median 8-year CD4 cell count in men reached the median reference value. CD4 cell count was nonlinearly associated with age. Middle-aged men showed higher median CD4 cell counts at 8 years compared to older and younger men when ART was started with a CD4 count of 350 or 500 cells/mm^3^. Women showed a similar but stronger pattern. Among those starting at a CD4 count of 500 cells/mm^3^, 45-year-old reference men (911 cells/mm^3^, 95% confidence interval: 889–933) and 51-year-old women (971 cells/mm^3^, 95% confidence interval: 932–1011) reached highest median 8-year CD4 cell counts. Trajectories of women aged 20 years at the start of ART were initially higher than those of older women during the first 2 years of ART, but flattened, whereas trajectories of women aged 37, 54, or 70 years at baseline kept increasing (see Figure SDC 8, Supplemental Digital Content, http://links.lww.com/QAI/B244). Similar results were obtained when analyses were restricted to those who reached <50 HIV copies per milliliter within 6 months from starting ART. CD4 cell count trajectories were also similar according to the starting year of ART (results not shown).

**FIGURE 2.**
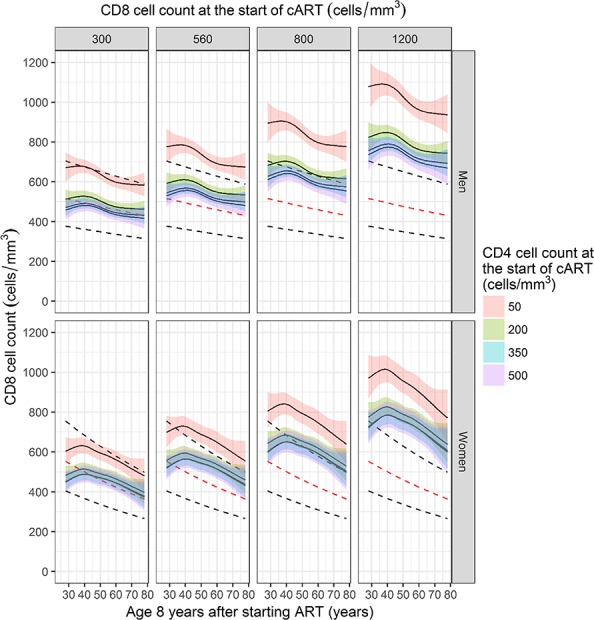
Median CD8 cell count after 8 years of virologically suppressive ART by age, gender, and CD4 and CD8 cell counts at the start of ART, for an average reference heterosexual individual born in Western Europe or North America and starting ART with 4.81 log_10_ copies per milliliter and random intercept and slopes equal to zero. Dashed lines show the upper, normal, and lower reference CD8 cell count values.

CD4 cell counts at 8 years were lower with increasing baseline CD8 cell counts until 400 cells/mm^3^, but the relation flattened off beyond 400 cells/mm^3^ (see Figure SDC 9, Supplemental Digital Content, http://links.lww.com/QAI/B244 in analysis additionally adjusted for baseline CD8 cell count in a subset of 37,305 individuals with CD8 cell counts available).

### CD8 Cell Count Trajectories

We used 374,985 CD8 cell count measurements from 37,305 individuals. The number of individuals with CD8 cell counts during virologically suppressive ART after 2, 4, 6, and 8 years was 18,316 (49%), 10,310 (28%), 5480 (15%), and 2525 (7%), respectively. The median CD8 cell count at 8 years was 765 cells/mm^3^ (IQR 558–1040). For those starting with 0–49 (410 individuals remaining in follow-up), 50–99 (n = 281), 100–199 (n = 655), 200–349 (n = 794), 350–499 (n = 242), and ≥500 CD4 cells/mm^3^ (n = 143), the median 8-year CD8 cell count was 800 (IQR 575–1100), 770 (566–1075), 774 (570–1006), 740 (540–1030), 751 (558–1050), and 810 (557–1072) cells/mm^3^, respectively.

Median CD8 cell counts at 8 years after the start of ART showed a similar downward trend with higher age as the trend observed in HIV-negatives (Fig. 2). This downward trend was not observed in men and women younger than 40 years, and median 8-year CD8 cell counts were similar across all ages below 40 years of age. Higher CD8 cell counts at the start were associated with higher CD8 cell counts at 8 years. Median 8-year CD8 counts were similar to median reference values when ART was started with a CD8 cell count of 300 cells/mm^3^ and a CD4 cell count of 200, 350, or 500 cells/mm^3^. Similar median CD8 cell counts at 8 years were reached for those starting with a CD4 count of 200, 350, or 500 cells/mm^3^. However, median 8-year CD8 cell counts were higher when ART was started with a CD4 count of 50 cells/mm^3^. The association between lower baseline CD4 cell counts and higher CD8 cell counts at 8 years starts from CD4 cell counts below approximately 250 cells/mm^3^ (see Figure SDC 10, Supplemental Digital Content, http://links.lww.com/QAI/B244).

Median CD8 cell count trajectories show a rapid decline in CD8 cell count during the first year when ART was started with a CD8 count of 800 or 1200 cells/mm^3^ and a more gradual decline after 1 year. CD8 cell counts remained higher than the median reference range during the first 8 years (see Figure SDC 11, Supplemental Digital Content, http://links.lww.com/QAI/B244).

### CD4:CD8 Ratio Trajectories

Median CD4:CD8 ratio at 8 years was 0.81 (IQR 0.57–1.11). The median 8-year ratio for those starting with 0–49, 50–99, 100–199, 200–349, 350–499, and ≥500 CD4 cell/mm^3^ was 0.63 (IQR 0.45–0.85), 0.66 (0.47–0.87), 0.76 (0.54–1.01), 0.89 (0.64–1.19), 1.05 (0.75–1.38), and 1.09 (0.88–1.50) cells/mm^3^, respectively.

**FIGURE 3.**
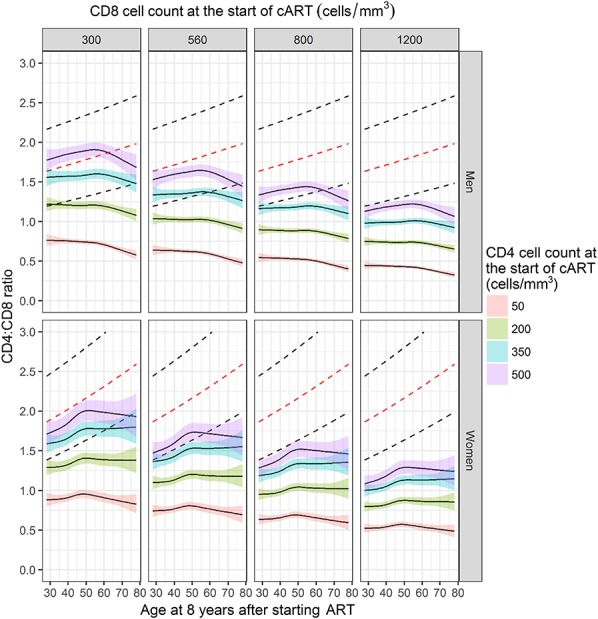
Median CD4:CD8 ratio at 8 years after the start of ART (95% confidence intervals in color) according to age at 8 years after the start of ART, gender, and CD4 and CD8 cell counts at the start of ART. Median ratios shown are those for an average reference individual in the heterosexual transmission risk group, born in Europe/North America, aged 37 years at the start of ART, and a plasma viral load of 4.81 log_10_ copies per milliliter at the start of ART and random intercept and slopes equal to zero. Dashed lines show the upper, normal and lower reference CD4:CD8 ratio in HIV-negative men and women.

Figure 3 shows that higher median CD4:CD8 ratios were reached when CD4 cell counts at the start of ART were higher and CD8 cell counts were lower (ie, the ratio at the start was higher). Among the combinations shown, median reference values were only reached in men younger than 60 years (at 8 years) who had started ART with the combination of a CD4 count of 500 cells/mm^3^ and CD8 count of 300 cells/mm^3^. Men and women with a CD4 count of 350 or 500 cells/mm^3^ at the start reached median CD4:CD8 ratios ≥1.0 at 8 years, irrespective of the CD8 cell count at the start, except in men older than 60 years of age at 8 years.

Median CD4:CD8 ratio trajectories continued to increase during the first 8 years of ART (see Figure SDC 12, Supplemental Digital Content, http://links.lww.com/QAI/B244). Ratio trajectories were higher in women compared with men.

### Association of Other Variables With Trajectories

The association between region of origin, transmission risk group, HIV RNA, smoking status, and HCV and CMV coinfection at the start of ART and CD4 and CD8 cell count and ratio trajectories and levels reached at 8 years is discussed in text and shown in figures (see Figures SDC 13–15, Supplemental Digital Content, http://links.lww.com/QAI/B244 and Table SDC 16, Supplemental Digital Content, http://links.lww.com/QAI/B244). In short, CD4 and CD8 cell count after 8 years of virologically suppressive ART were higher in smokers compared with nonsmokers. Median trajectories of CD4 cell count and the CD4:CD8 ratio were lower in individuals infected by IDU compared with non-IDU. CMV-negative individuals showed a stronger decrease in CD8 cell count in the first year after starting ART compared with CMV-positive individuals, and because CD4 cell count trajectories were fairly similar, CD4:CD8 ratio trajectories in CMV-positive individuals were lower compared with those in CMV-negative individuals.

## DISCUSSION

As ART is now both recommended and often started at high CD4 cell counts, we investigated whether median trajectories of CD4 and CD8 cell count and the CD4:CD8 ratio reach median reference values when ART is started with high (eg, 500 cells/mm^3^) and lower CD4 cell counts (such as 350, 200, and 50 cells/mm^3^). During 8 years of virologically suppressive ART, median reference CD4 cell counts (about 800 cells/mm^3^) were reached only by men starting at high CD4 cell counts ≥500 cells/mm^3^. Despite virologically suppressive ART, CD8 cell count trajectories converged after 2 years to a stable level above the median reference value. Median reference CD8 cell counts were only reached when ART was started at low CD8 cell count (such as 300 cells/mm^3^). As a result of persisting high CD8 cell counts, median reference CD4:CD8 ratios were not reached, even when ART was started at high CD4 cell counts.

Measurements after interruption of ART of more than 2 weeks and after a confirmed HIV RNA >400 copies per milliliter were not included in the analysis. Our results are, therefore, not generalizable to all individuals who start ART but rather provide an estimate of the maximum capacity of the immune system to restore CD4 and CD8 cell counts and their ratio during long-term virologically suppressive ART. Our selection of individuals, therefore, will also include some immunological nonresponders, individuals with nonperfect adherence, women during pregnancy, and individuals with certain comorbidities or comedication that may impact CD4 and CD8 cell count trajectories. Furthermore, generalizability may be slightly impaired if those that interrupt ART or with virological failure have different CD4 or CD8 cell count trajectories before interruption or failure.

Median reference CD4 cell counts were only reached within 8 years in more than 50% of men when ART was started with high CD4 cell counts (such as 500 cells/mm^3^). By definition, approximately 50% of individuals will have had a pre–HIV-infection CD4 cell count below the population median, and some even below 500 cells/mm^3^. Hence, those who start ART with a CD4 count more than 500 cells/mm^3^ are already a selected subgroup that probably had pre-HIV CD4 counts above the population median. Likewise, those who had lower-than-average CD4 counts before they acquired HIV are more likely to start ART with low CD4 counts. Ideally, one would like to compare CD4 cell counts during virologically suppressive ART with an individual's pre–HIV-infection CD4 cell count level, but we did not have data on pre-HIV infection CD4 cell counts for the individuals in our study. The level of the first postseroconversion CD4 cell count has been shown to be predictive for the CD4 cell count recovery after the start of ART.^35^ However, also the timing of seroconversion and postseroconversion CD4 cell count were mostly unknown in our data.

The highest CD4 cell counts after 8 years of virologically suppressive ART were observed for women aged 51–52 years. Although in women we observed a peak CD4 cell count response during middle age at all levels of CD4 cell count at the start, in men, a similar peak was only observed when ART was started at CD4 counts of 500 cells/mm^3^, but not at lower CD4 cell counts. A similar nonlinear age–CD4 response pattern was found in a recent study.^36^ Because we did not observe such a trend in the HIV-negative population, it is not clear whether an underlying biological process contributes to our findings. Adherence to ART has been shown to be less in younger individuals,^37^ and differences in compliance may exist even when all HIV RNA measurements are below the limit of detection. Poorer adherence may explain the smaller increases in CD4 cell count after 2–8 years after the start of ART when ART was started at the age of 20 years compared with middle-aged men and women. We aimed to select more adherent individuals in the sensitivity analysis with a stricter definition of virologically suppressive ART but results were similar to the main analysis. The peak in CD4 response in women coincides with the mean age of natural menopause, but studies have found no evidence for a difference in CD4 cell count response after ART initiation between premenopausal and postmenopausal women with a virological response.^38,39^

A recent study found that CD8 cell count trajectories, adjusted for baseline CD4 cell count but not for baseline CD8 cell count, were very similar for those starting ART with a CD4 count of ≥200 cells/mm^3^ but long-term CD8 cell count trajectories were higher with lower baseline CD4 count when ART was started with counts <200 cells/mm^3^.^29^ We show that reaching higher long-term CD8 cell counts is not only associated with lower baseline CD4 cell count but also with younger age and higher baseline CD8 cell count. CD8 cell counts at 8 years were lower with older age, similar, albeit at a higher value, compared to the trend in HIV-negative individuals. The association between higher baseline CD4 cell counts and lower CD8 cell count trajectories is counterbalanced by the association between higher baseline CD8 cell counts. Higher baseline CD8 cell counts usually accompany higher baseline CD4 cell counts and are associated with higher CD8 cell count trajectories. Together, this results in long-term CD8 cell counts that are similar when ART is started with a CD4 count of 200 cells/mm^3^ or higher. Lack of normalization of CD8 cell counts in HIV-positive individuals was also observed in a recent study by the Danish HIV Cohort.^40^ Elevated CD8 cell count levels are suggestive of ongoing residual immune activation, and residual HIV viremia, coinfections (such as CMV^41^), microbial translocation, loss of immunoregulatory responses, and hypercoagulability are all thought to contribute.^42,43^

When ART was started at CD4 counts above 200 cells/mm^3^ in men and 250 cells/mm^3^ in women, median CD4 cell count trajectories reached ≥500 cells/mm^3^ within 8 years of virologically suppressive ART. When ART was started at a CD4 count of 350 or 500 cells/mm^3^, median ratios >1 were reached within 8 years. These cutoffs are frequently used to identify individuals at increased risk of morbidity and/or mortality. However, there continues to be an association of a lower risk of AIDS and death with a higher CD4 cell count, even above 500 cells/mm^3^.^44,45^ For the CD4:CD8 ratio and CD8 cell count, these associations are less clear cut. Several studies have suggested that the CD4:CD8 ratio, independent of CD4 cell count, predicts time to death and non–AIDS-defining morbidity.^22–24,46^ In addition, both low CD8 cell count in the first year after starting ART and increased CD8 cell counts >1500 cells/mm^3^ at 10 years after the start of ART have been associated with mortality.^40^ By contrast, a recent ART-CC study found only a small effect of CD8 cell counts and no significant effect of the CD4:CD8 ratio on all-cause mortality.^47^ It may be that associations between either CD8 cell count or CD4:CD8 ratio and clinical outcome are only limited to specific causes of morbidity or death.

A limitation of our study was that analyses including data on CD8 cell counts, smoking, and HCV and CMV coinfection were performed in different subsets of individuals, which limits the interpretation of the results because we did not adjust for all covariates at the same time. Furthermore, there were few HIV-negative people older than 65 years. Therefore, comparisons with median HIV-negative values beyond 65 years are mostly based on extrapolation. Another limitation is that both HIV-negative cohorts were from a Western European population, whereas CD4 cell counts are generally reported to be lower in sub-Saharan African populations.^48^ In our study, CD4 and CD8 cell count and CD4:CD8 ratio trajectories in HIV-positive individuals from sub-Saharan Africa were all somewhat lower compared with those from Western Europe or North America. Whether these differences translate into differential risk of morbidity or mortality is difficult to investigate because of other socioeconomic and psychosocial differences.

## CONCLUSION

Starting ART with a CD4 cell count of ≥500 cells/mm^3^ makes reaching a CD4 cell count comparable with those seen in HIV-negative individuals more likely. However, even when ART is started with a high CD4 cell count, median CD4:CD8 ratio trajectories remained below the reference levels of HIV-negative individuals because of persisting high CD8 cell counts. To what extent these subnormal immunological responses affect specific clinical endpoints requires further investigation.

## Supplementary Material

SUPPLEMENTARY MATERIAL
