# Supplementary material for: Determinants of Restoration of CD4 and CD8 Cell Counts and Their Ratio in HIV-1–Positive Individuals With Sustained Virological Suppression on Antiretroviral Therapy
Source: J Acquir Immune Defic Syndr. 2018 Dec 3;80(3):292–300. doi: 10.1097/QAI.0000000000001913 (PMC6392208; doi:10.1097/QAI.0000000000001913)
Supplement: SUPPLEMENTARY MATERIAL [file qai-80-292-s001.docx]

**SDC 1**

**HIV-negative study participants**

We estimated median reference ranges for CD4 and CD8 cell count, and the CD4:CD8 ratio using data obtained from two sources. One consisted of healthy individuals from the background population to the Danish HIV cohort recruited from healthy staff and blood and stem-cell donors. Secondly, we included HIV-negative participants from the AGE_h_IV cohort study, recruited either at the STI clinic of the Amsterdam Public Health Service or the existing Amsterdam Cohort Studies on HIV/AIDS [26]. Each individual contributed one CD4 and CD8 cell count measurement.

Linear regression was used to obtain reference values for CD4 cell counts, CD8 cell counts and the CD4:CD8 ratio according to age and gender. Using the boxcox function in the R package MASS, residuals in the linear regression analysis of CD4 cell counts were found to best comply with normality assumptions when counts were 5^th^ root transformed, whilst CD8 cell counts and the CD4:CD8 ratio were log transformed. The age variable was modeled linearly as well as via restricted cubic splines with varying (3-5) number of knots. Differences between models were assessed using the Akaike Information Criterion (AIC). We tested for differences between cohorts by including cohort as a covariate in the models. We used the estimated 2.5 and 50 prediction percentiles as lower and median reference values in the analyses of immunological restoration after starting ART in HIV infected individuals.

**Results**

CD4 and CD8 cell count and CD4:CD8 ratio measurements were available for 2,309 HIV-negative individuals, 1,797 Danish blood and stem cell donors and healthy staff, and 512 participants in the AGE_h_IV cohort study. Supporting information table S1 shows the distribution according to gender and age. Participants in the Danish blood donor study were mostly aged between 18 and 45 years whilst all of the HIV-negative participants in the AGE_h_IV study were 45 years of age or older.

Median CD4 cell count declined with increasing age. The decline in CD4 cell count with increasing age was greater in women (p=0.03), than in men (p=0.45). Median CD8 cell count also declined with increasing age whilst the median CD4:CD8 ratio increased with increasing age. There was strong evidence for a decline in CD8 cell count and an increase in CD4:CD8 ratio with increasing age in both men (p=0.0005 and p=0.0008, respectively) and women (both p<0.0001). There was no evidence of a difference in CD4 or CD8 cell counts between the two cohorts. There was some evidence that the CD4:CD8 ratio in men from the Danish cohort was higher than that in men from the AGE_h_IV cohort (p=0.02).

**SDC 2**

**HIV-positive study participants**

Cohorts included in this paper were AGEhiV the Netherlands, the Cohort of the Spanish HIV Research network (CoRIS), Spain; the French Hospital Database on HIV (FHDH); the Italian Cohort of Antiretroviral-naïve patients (ICONA); the Swiss HIV Cohort Study (SHCS); the AIDS Therapy Evaluation project, Netherlands (ATHENA); The Multicenter Study Group on EuroSIDA; the Aquitaine Cohort; the Royal Free Hospital Cohort; the South Alberta Clinic Cohort; The Danish HIV Cohort Study, Denmark; HAART Observational Medical Evaluation and Research (HOMER) Cohort, Canada; HIV Atlanta Veterans Affairs Cohort Study (HAVACS), US; Koln/Bonn Cohort, Germany; Osterreichische HIV-Kohortenstudie (OEHIVKOS), Austria; Proyecto para la Informatizacion del Seguimiento Clinico-epidemiologico de la Infeccion por HIV y SIDA (PISCIS), Spain; University of Alabama 1917 Clinic Cohort, USA; University of Washington HIV Cohort, US; VACH, Spain; Veterans Aging Cohort Study (VACS), US; and Vanderbilt-Meherry, US.

**SDC 3**

**Statistical analysis**

While ethnicity was known in 40% of individuals, region of birth was known in 84% of individuals and was therefore included in the analyses. In individuals with missing region of birth, information on ethnicity could be used to reclassify 353 individuals as having been born in Caribbean/South America, and 313 as having been born in Sub Saharan Africa. In addition, 4,584 individuals with white ethnicity were reclassified as having been born in Europe/North America and 988 with black ethnicity as having been born in Sub Saharan Africa (their CD4 cell count trajectories were similar to those born in Sub Saharan Africa). The remaining 3,445 individuals were classified into other/unknown region of birth.

**Interaction and spline modeling**

The association between continuous covariates and the trajectories of CD4 and CD8 cell counts and their ratio were modeled via splines, with knots approximately at the 2.5^th^, 27.5^th^, 50^th^, 72.5^th^ and 97.5^th^ percentiles (knots for CD4 cell count at the start were chosen at 10, 160, 260, 375 and 740 cells/mm^3^, knots for CD8 cell count at the start at 190, 560, 830, 1200 and 1600 cells/mm^3^ and knots for age at the start of ART at 22, 32, 39, 47 and 63 years). Log_10_ transformed plasma viral load was included linearly. Furthermore, we allowed the effect of region of birth on the time trend to differ by gender, and we allowed for three-way interactions between the time trend and i) age and CD4 cell count at the start of ART, ii) gender and age, and iii) gender and CD4 cell count at the start of ART. In these three-way interaction terms, time was modeled as a restricted cubic spline with knots at 0, 0.1, 0.25, 3, and 7.5 years, age at the start of ART as a restricted cubic spline with knots at 22, 39, and 63 years, and CD4 cell count at the start of ART as a restricted cubic spline with knots at 10, 260, and 740 cells/mm^3^.

F**igure SDC4A-C**: Median (solid line), and 25^th^ and 75^th^ percentile (dashed lines) of A: CD4 and B: CD8 cell count and C: CD4:CD8 ratio in 1253 HIV-negative men (blue lines) and 1056 women (red lines). Individual observations are shown in blue dots for men and red dots for women.


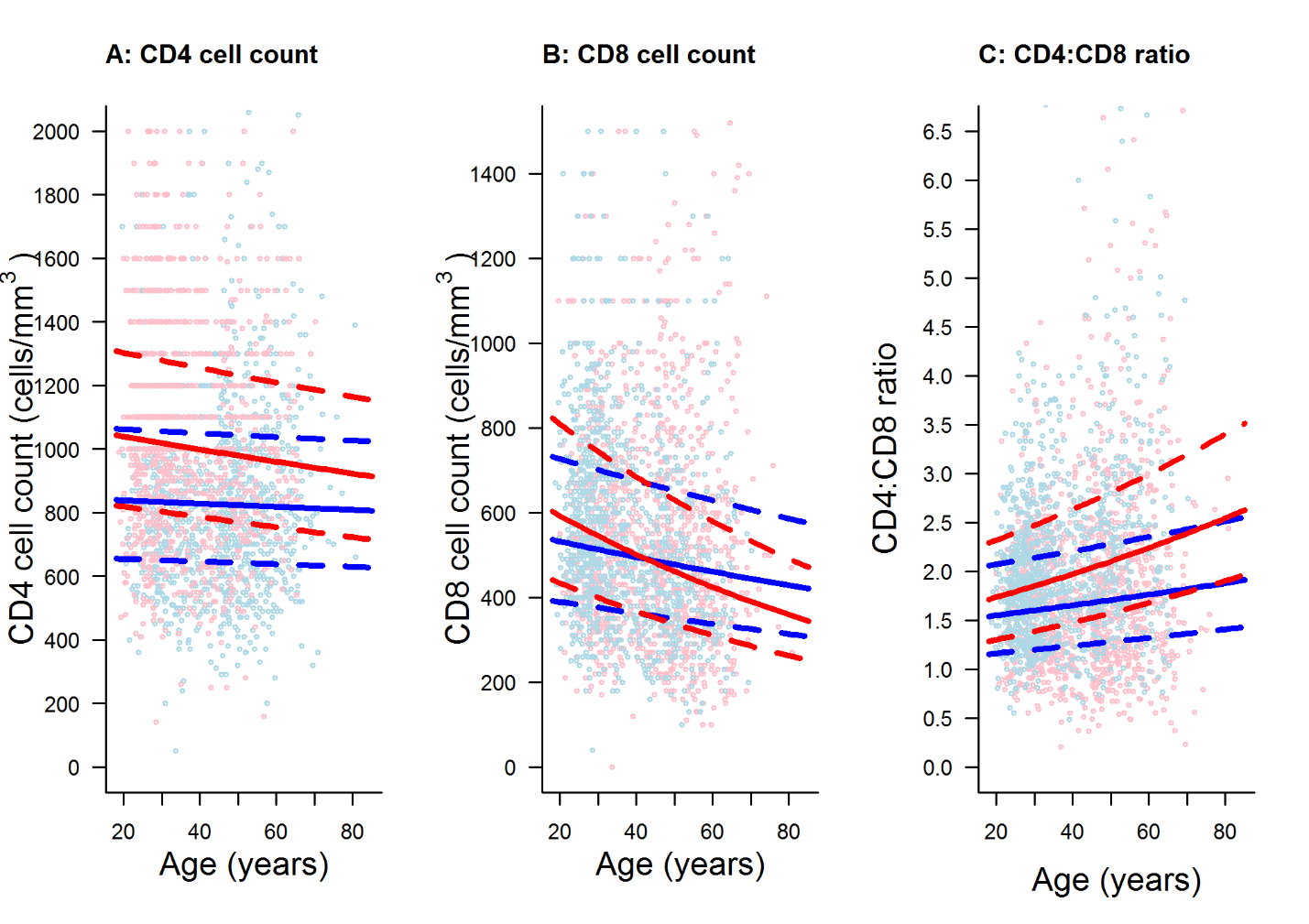


F**igure SDC5A-C**: Median CD4 cell counts (solid lines) and 95% confidence intervals (dashed lines) in HIV-negative men (dark blue) and women (dark red) obtained using linear regression models with age included as restricted cubic spline with 3 (A), 4 (B), and 5 (C) knots. In each figure we also show the estimates obtained using models that include age linearly on the transformed scale (blue lines for men and red lines for women). The model with 3 knots fitted the data significantly better than the linear (p=0.008), the model with 4 knots better than the 3-knot model (p=0.001) and the 5-knot model better than the-4 knot model (p=0.004).

3 knots (at 23, 39, and 62 years) 4 knots (at 23, 31,47 and 62 years).

F**igure SDC6A-C**: Median CD8 cell counts (solid lines) and 95% confidence intervals (dashed lines) in HIV-negative men (dark blue) and women (dark red) obtained using linear regression models with age included as restricted cubic spline with 3 (A), 4 (B), and 5 (C) knots. In each figure estimates were obtained using models with age included as a linear predictor on the transformed scale are also shown (blue lines for men and red lines for women). The fit of the linear model was not significantly different from the 3 and 4 knot models and was slightly better for the 5 knot model compared to the linear model (p=0.03).

3 knots (at 23, 39, and 62 years) 4 knots (at 23, 31, 47 and 62 years).

5 knots (at 23, 35, 50, 55, and 62 years)

**Figure SDC7A-C**: Median CD4:CD8 ratio (solid lines) and 95% confidence intervals (dashed lines) in HIV-negative men (dark blue) and women (dark red) obtained using linear regression models with age included as a restricted cubic spline with 3 (A), 4 (B), and 5 (C) knots. In each figure estimates obtained using models including age as a linear predictor on the transformed scale are also shown (blue lines for men and red lines for women). The fit of the linear model was not significantly different from the 3 and 4 knot models and was slightly better for the 5 knot model compared to the linear model (p=0.02).

3 knots (at 23, 39, and 62) 4 knots (at 23, 31,47 and 62).

5 knots (at 23, 35, 50, 55, and 62)

**Figure SDC8.** Median CD4 count trajectories (95% confidence intervals in colour) during virologically suppressive ART according to age at the start of ART, gender, and baseline CD4 cell count at the start. Trajectories shown are those for an average individual (in the heterosexual transmission risk group, born in Western Europe/North America , starting ART between 2004 and 2006 with a baseline plasma viral load of 4.81 log_10_ copies/ml and with a random intercept and slopes equal to zero). Dashed lines show the estimated lower and median reference CD4 cell count.

**Figure SDC9.** Median CD4 cell count at eight years of virologically suppressive ART (95% CI’s in colour) according to CD8 cell count at baseline for an average individual (37 year old heterosexual male, born in Western Europe/North America with a plasma viral load of 4.81 log_10_ copies/ml at the start of ART with a random intercept and slopes equal to zero). Dashed lines show the lower and median reference CD4 cell count for a 37-year old male.

F**igure SDC10.** Median CD8 cell count after eight years of virologically suppressive ART, by CD4 count at the start of ART, gender, age eight years after the start, and CD8 cell count at the start of ART for an average individual (born in Western-Europe or North-America, in the heterosexual transmission risk group and starting ART with HIV RNA 4.81 log_10_ copies/ml and with a random intercept and slopes equal to zero). Dashed lines show the lower, median and upper reference CD8 cell count for various ages.**
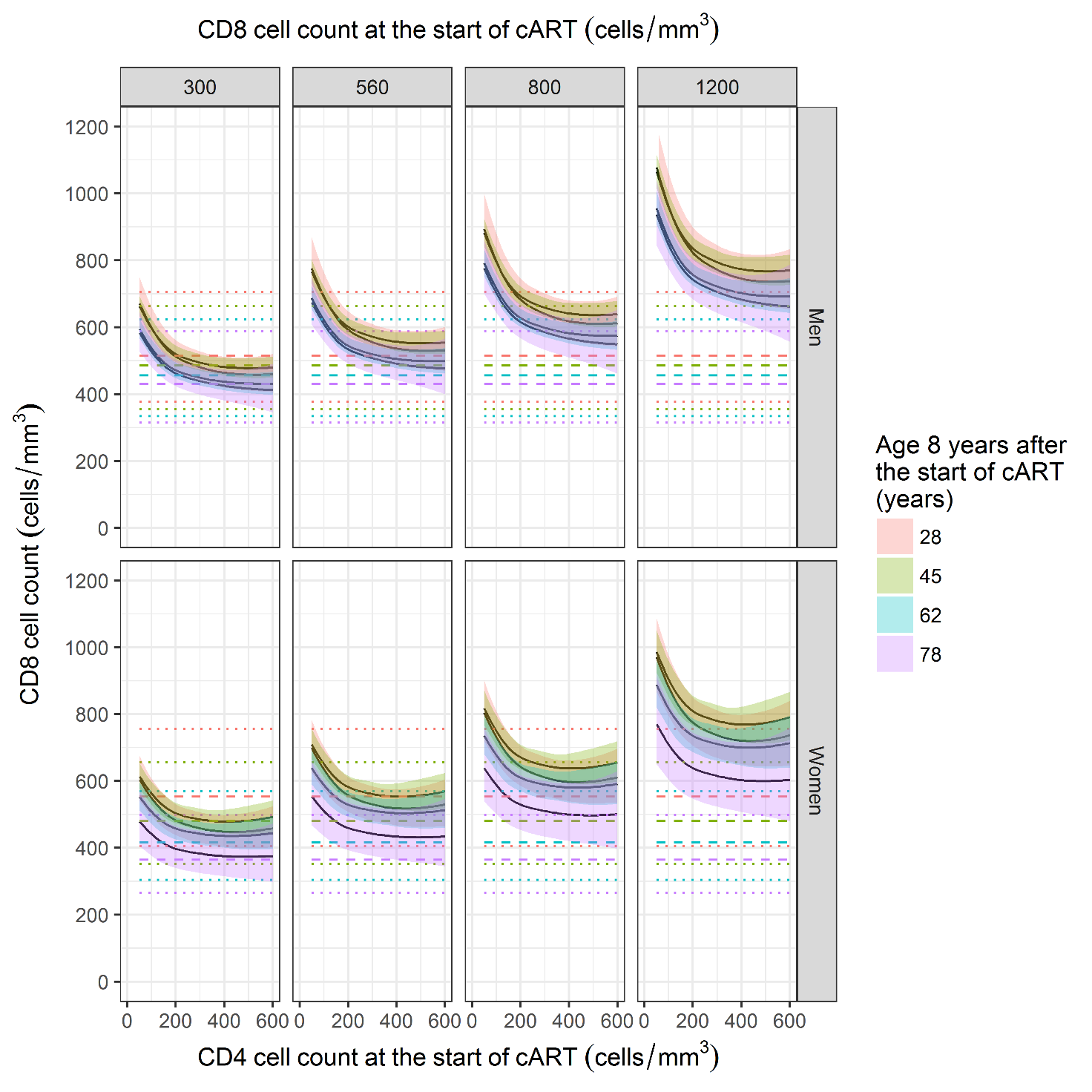
**

**Figure SDC11.** Median CD8 cell count trajectories during eight years of virologically suppressive ART by CD4 and CD8 cell count and age at the start of ART for an average reference individual (male heterosexual starting ART with a plasma viral load of 4.81 log_10_ copies/ml, born in Western Europe or North America and random intercept and slopes equal to zero). Shaded coloured areas are 95% confidence intervals. Dashed lines show the upper, normal and lower reference CD8 cell counts for HIV-negative subjects of the same gender and age.


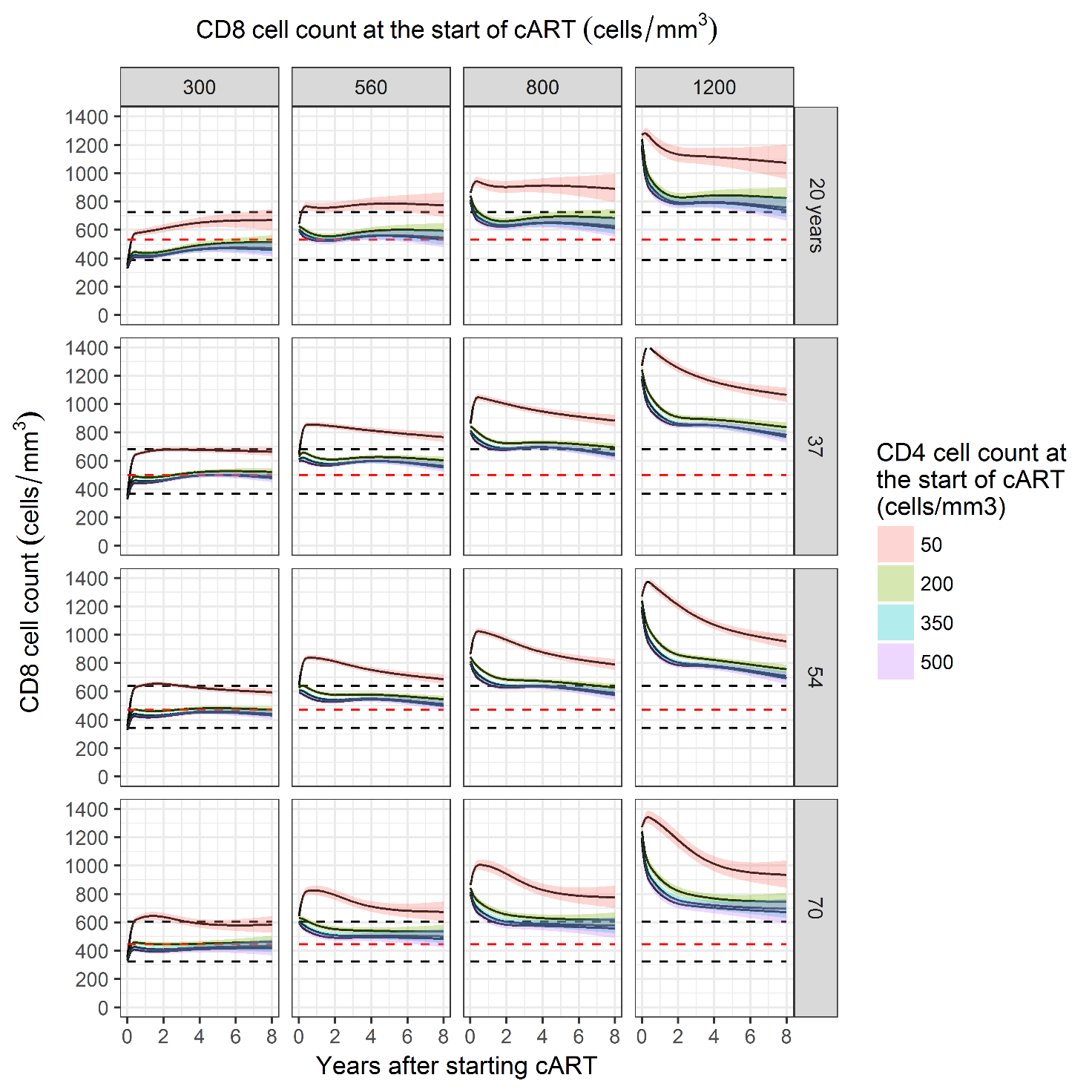


**Figure SDC12.** Median CD4:CD8 ratio trajectories (95% confidence intervals in colour) during virologically suppressive ART according to gender, and CD4 (vertical axis) and CD8 (upper horizontal axis) cell count at the start of ART. Trajectories shown are those for an average reference individual in heterosexual transmission risk group, born in Europe/North America, 37 years of age at the start of ART, and a plasma viral load of 4.81 log_10_ copies/ml at the start of ART and random intercept and slopes equal to zero. Dashed lines show the lower and normal reference CD4:CD8 ratio.


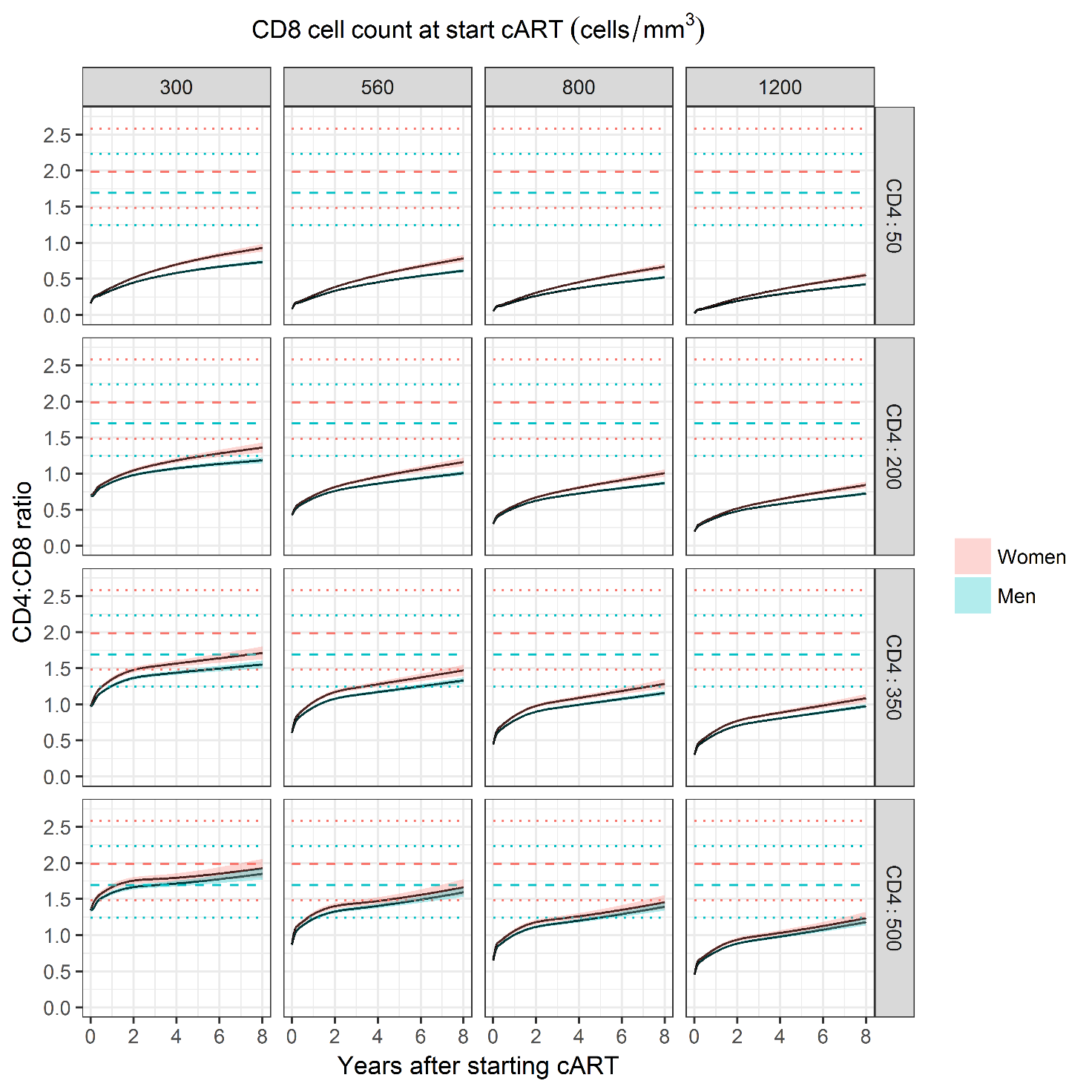


Supplemental Digital Content 13A-F show the effect of other variables on CD4 cell count response. Increases in CD4 cell count during eight years of virologically suppressive ART were smaller in those infected through IDU (p<0.0001 compared to non-IDU), in individuals born in Sub Saharan Africa (interaction time and region of birth overall p<0.0001), in individuals with lower plasma viral load at the start of ART (p<0.0001) in non-smokers (p<0.0001), and in HCV-positive individuals (p<0.0001). Data on ribavirin treatment was available for 13 of the 14 cohorts included in the HCV analysis. Ribavirin treatment was given for some period during the first eight years of ART to 754 individuals out of 4733 HCV-positive individuals (16%) from these 13 cohorts. CD4 cell count trajectories were similar in CMV-negative and positive individuals, although the p-value for the difference in trajectory was 0.0002. CD4 cell count trajectories according to start year of ART were similar (results not shown).

**Figure SDC13A-F**. Median CD4 cell count trajectories during virologically suppressive ART (95% confidence intervals in colour) according to A) transmission risk group, B) region of birth, C) plasma viral load at the start of ART, D) smoking status, E) HCV, and F) CMV co-infection, for an average individual (37-year old male, and unless otherwise stated in the legend, not infected through IDU, born in Western Europe/North America, starting ART with a HIV RNA of 4.81 copies/ml and a CD4 count of 350 cells/mm^3^ and with a random intercept and slopes equal to zero). Dashed lines show the lower and median reference CD4 cell count.

IDU: intravenous drug use, SSA: Sub Saharan Africa, CAR/SA: the Caribbean/South America, EUR/NA: Western Europe/North America, HCV: hepatitis C virus, CMV: cytomegalovirus


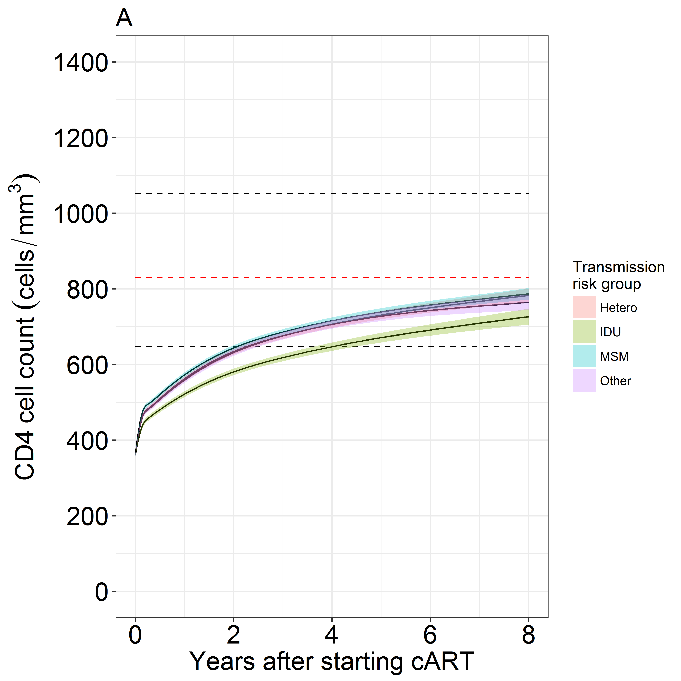

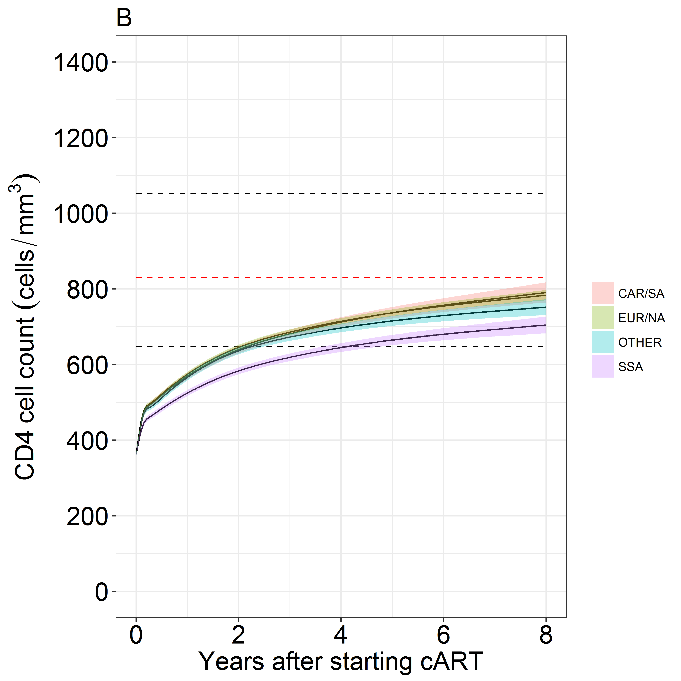

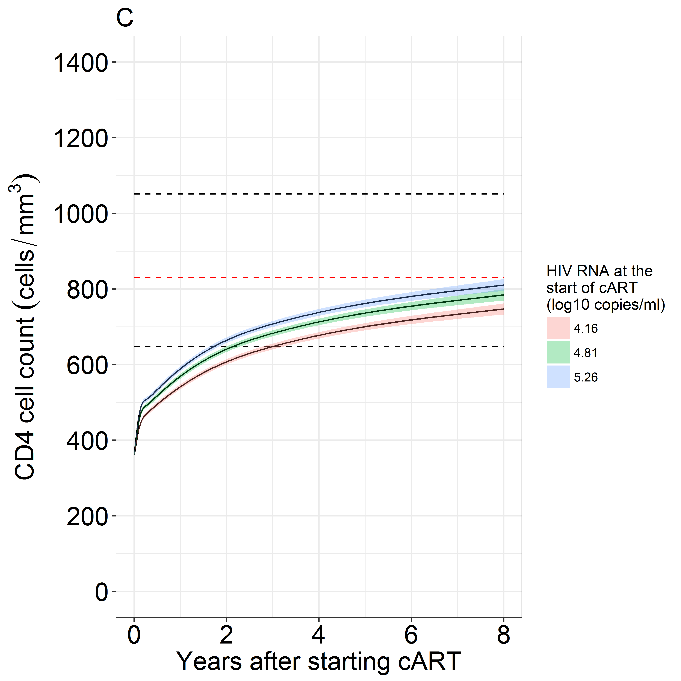

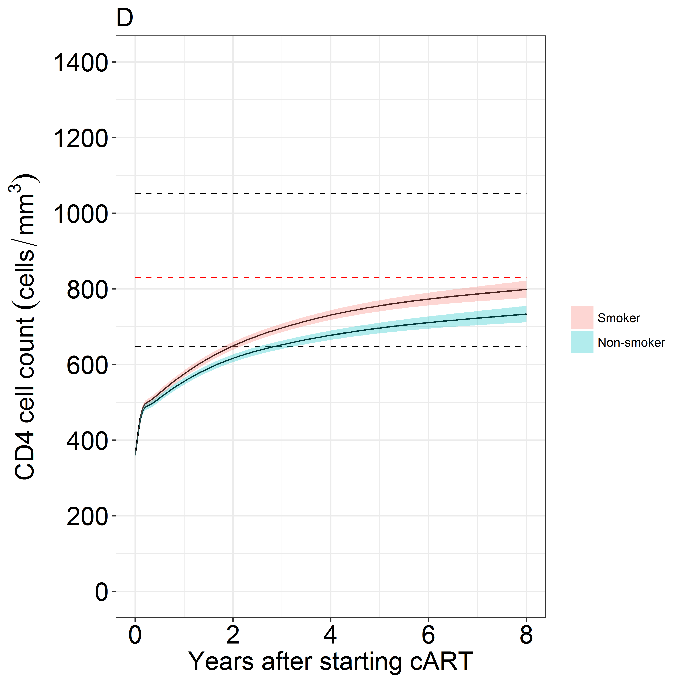

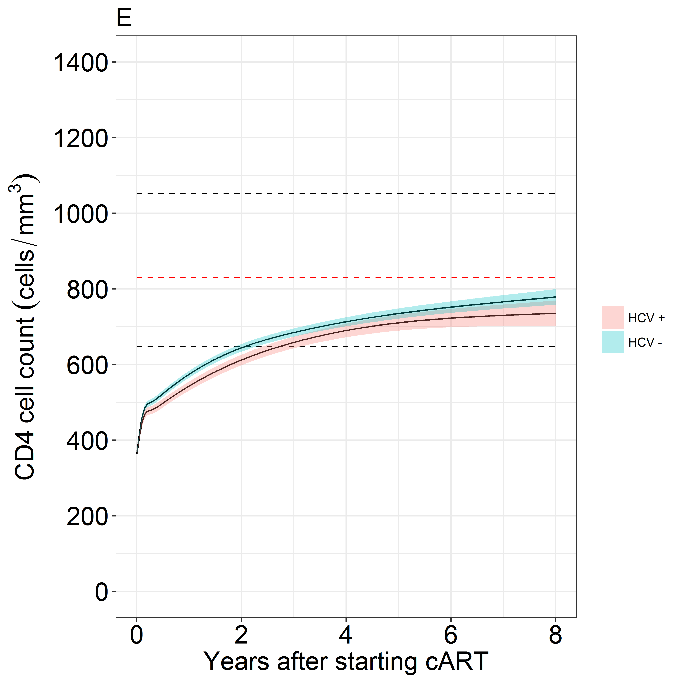

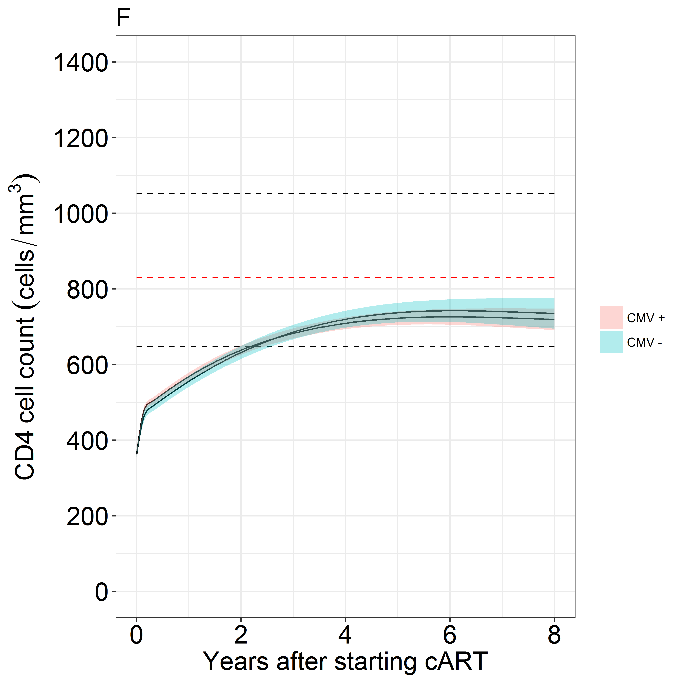


Supplemental Digital Content 14A-F show that median CD8 cell trajectories were higher in MSM than in those in the IDU and heterosexual risk groups (overall p-value for interaction between time and transmission risk group p<0.0001). Individuals born in Sub-Saharan Africa showed lower CD8 cell count trajectories compared to those born elsewhere (overall p-value for region of birth <0.0001). CD8 cell count trajectories were higher when HIV RNA at the start of ART was higher (p<0.0001). CMV-negative individuals showed a stronger decrease in CD8 cell count in the first year after starting ART compared to CMV-positive individuals (p<0.0001). HCV-negative and positive individuals showed largely similar trajectories, although statistically significantly different (p<0.0001). Median CD8 cell count trajectories were higher in smokers than in non-smokers (p<0.0001).

**Figure SDC14A-F**. Median CD8 cell count trajectories during eight years of virologically suppressive ART for an average individual (37-year old male, and unless otherwise stated in the legend, in the heterosexual transmission risk group, born in Western Europe/North America, starting ART with a HIV RNA of 4.81 copies/ml, a CD4 count of 350 cells/mm^3^ and a CD8 count of 800 cells/mm^3^ and with a random intercept and slopes equal to zero) according to transmission risk group (A), region of birth (B), HIV RNA (C), smoking status (D), HCV status (E), and CMV status (F). Shaded coloured areas are 95% confidence intervals. Dashed lines show the lower, median and upper reference CD8 cell counts.

IDU: intravenous drug use, SSA: Sub Saharan Africa, CAR/SA: the Caribbean/South America, EUR/NA: Western Europe/North America, HCV: hepatitis C virus, CMV: cytomegalovirus.


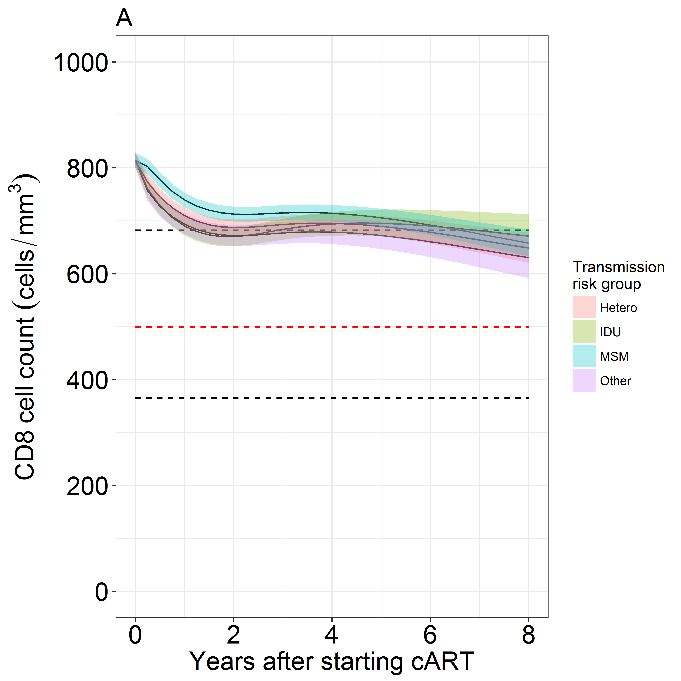

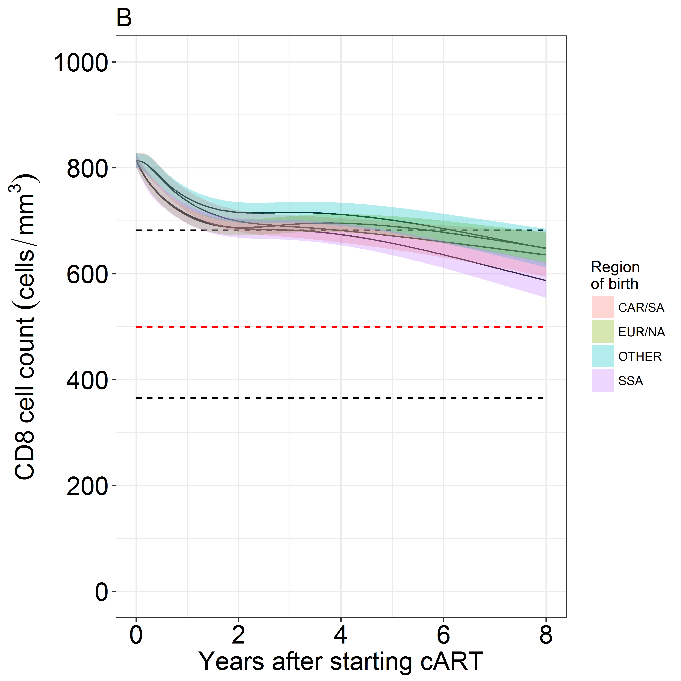

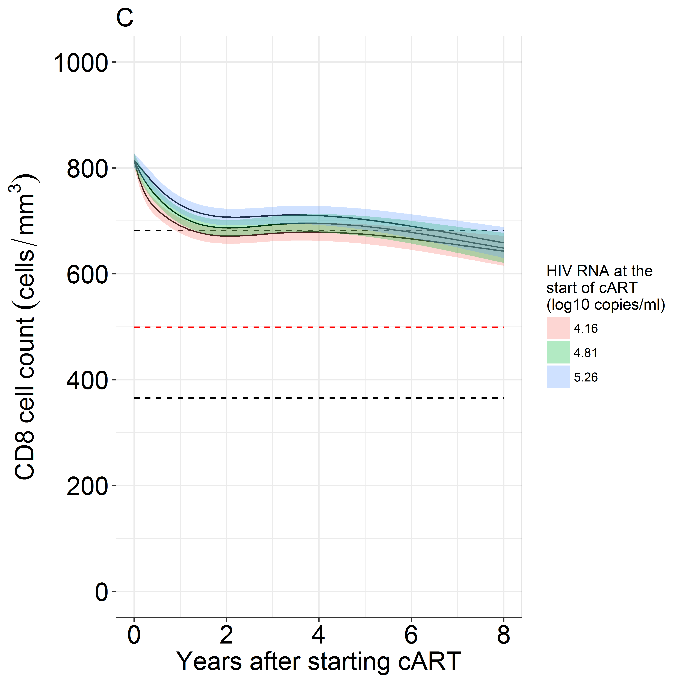

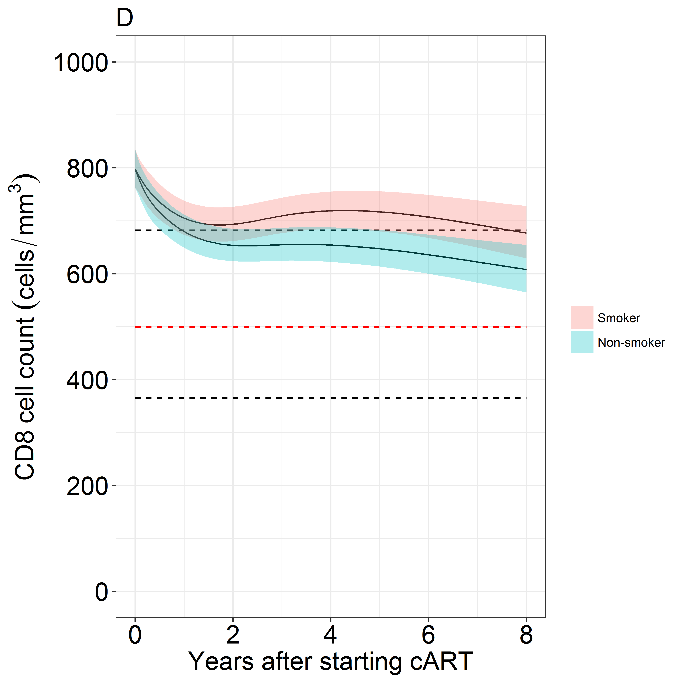

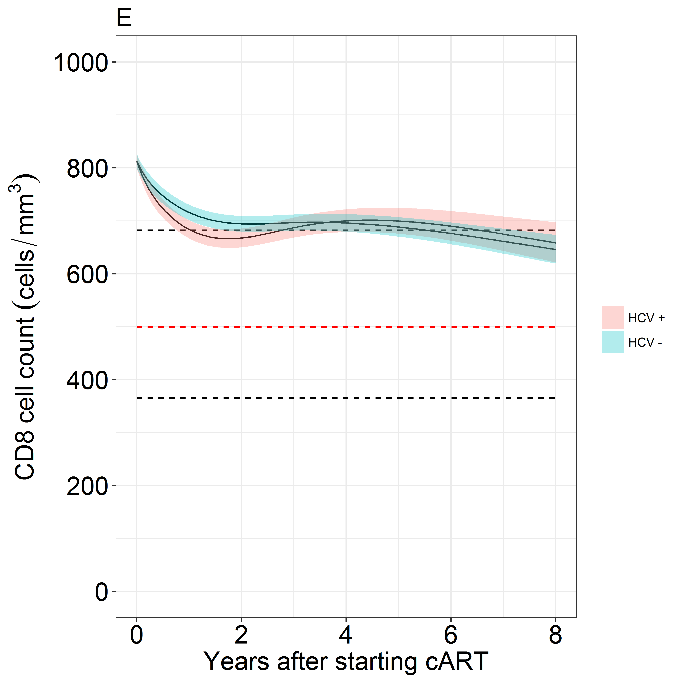

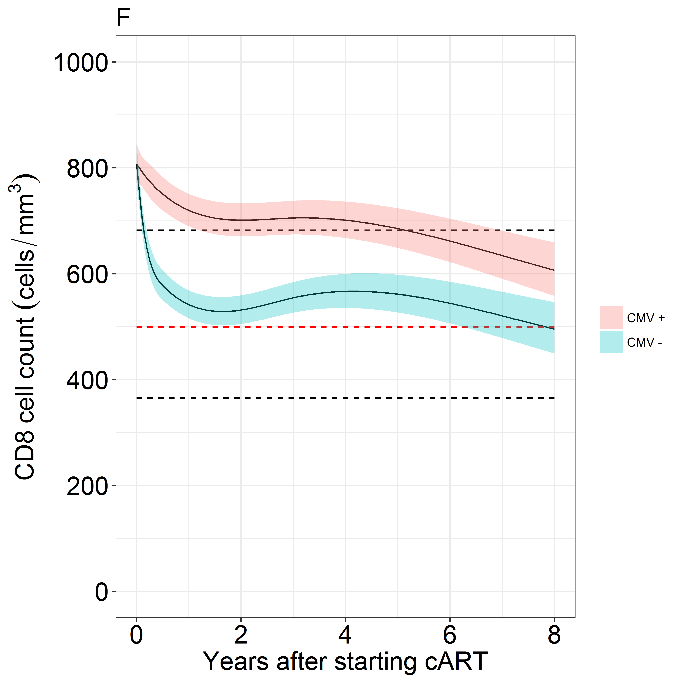


Supplemental Digital Content 15 shows that median CD4:CD8 ratio trajectories were somewhat lower in men from Sub Sahara Africa compared to men from Western Europe/North America and the Caribbean/South America (interaction between time and region of birth p<0.0001). Furthermore, supporting information figure 10A, C, D, E, and F shows that ratios were lower in individuals in the IDU transmission risk group compared to those in other risk groups (interaction between time and transmission risk group, p<0.0001), in individuals with HCV co-infection at the start of ART (p<0.0001), in individuals with a CMV co-infection at the start of ART (p<0.0001), and in those with lower plasma viral load at the start of ART (p<0.0001). CD4:CD8 trajectories were similar for smokers and non-smokers (p=0.26).

**Figure SDC15A-F.** Median CD4:CD8 ratio trajectories (95% confidence intervals in colour) during virologically suppressive ART according to transmission risk group (A), region of birth (B), HIV RNA (C), smoking status (D), HCV co-infection (E), and CMV co-infection (F) at the start of ART. Trajectories are shown for an average individual (37-year old male, and unless otherwise stated in the legend, in the heterosexual transmission risk group, born in Western Europe/North America, starting ART with a HIV RNA of 4.81 copies/ml, a CD4 count of 350 cells/mm^3^ and a CD8 count of 800 cells/mm^3^ and with a random intercept and slopes equal to zero). Dashed lines show the estimated median and lower reference CD4:CD8 ratio.

IDU: intravenous drug use, SSA: Sub Saharan Africa, CAR/SA: the Caribbean/South America, EUR/NA: Western Europe/North America, HCV: hepatitis C virus, CMV: cytomegalovirus.


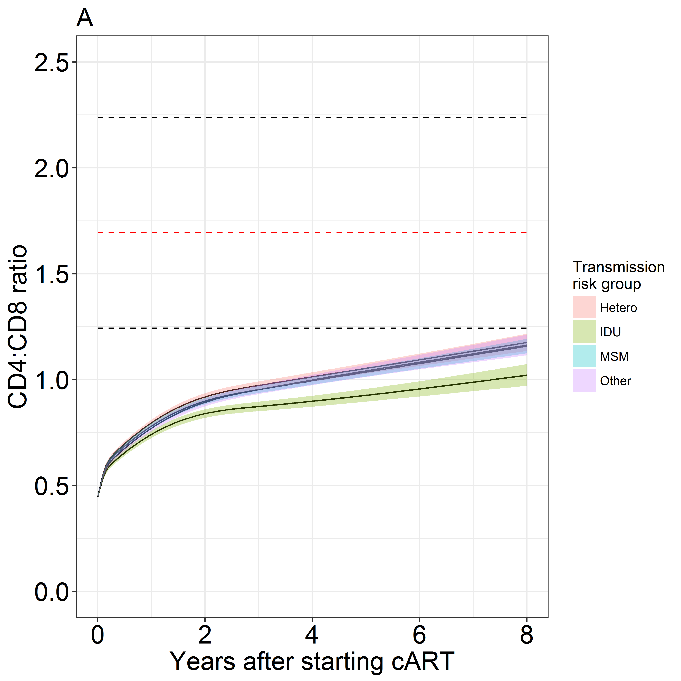

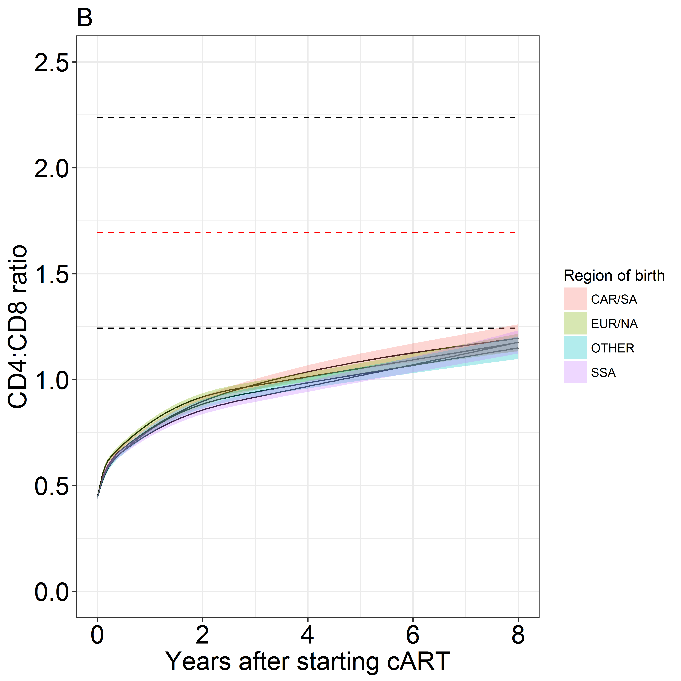


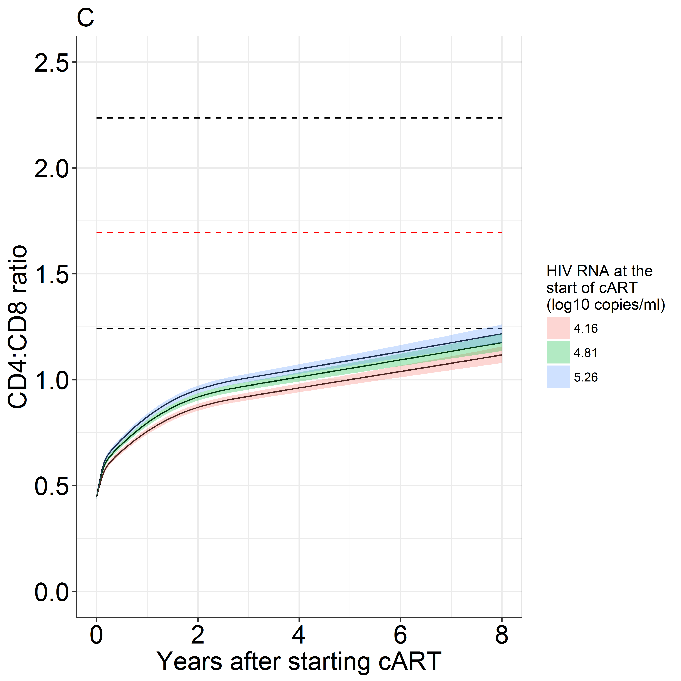

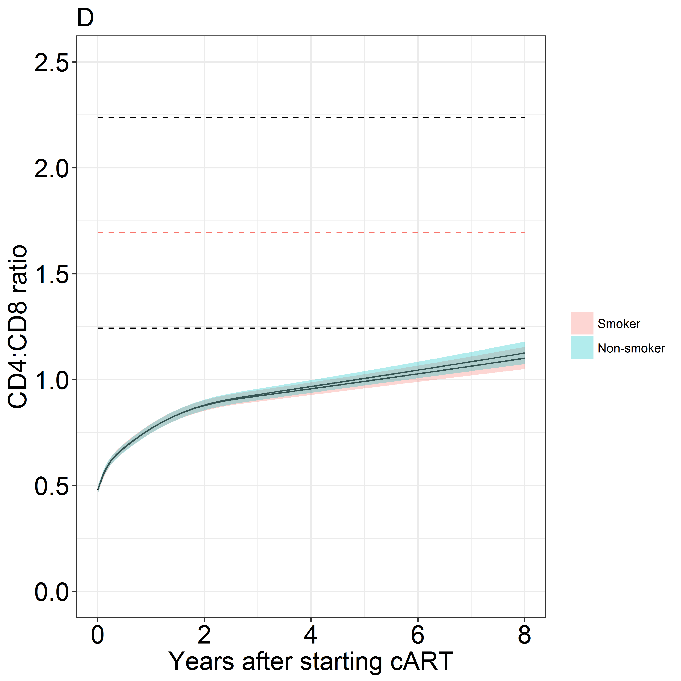


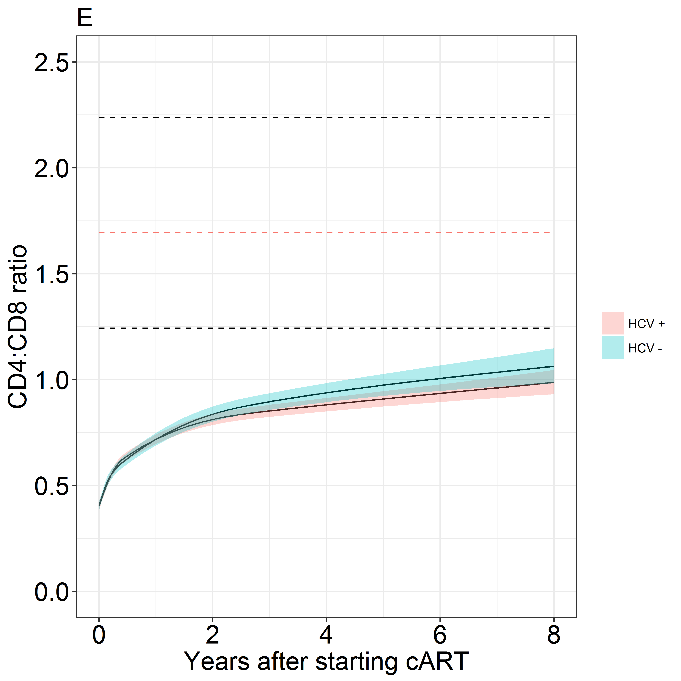

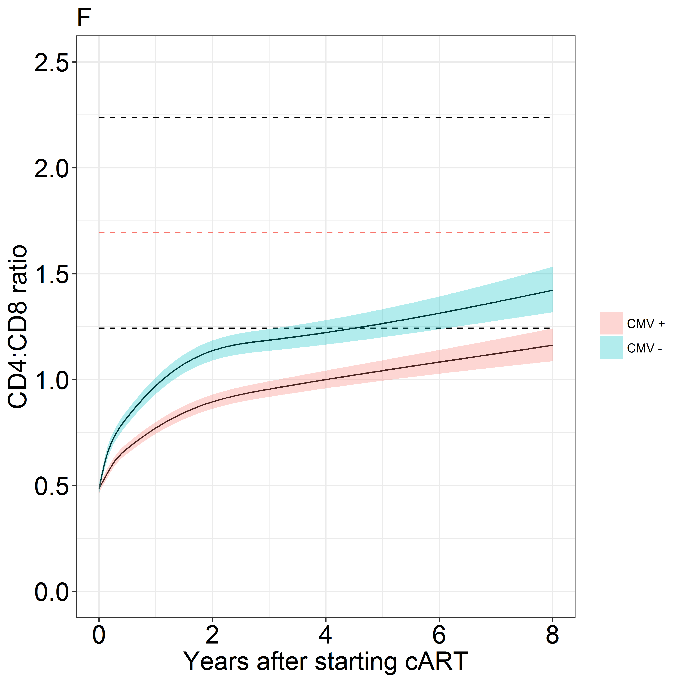


**Table SDC16.** Estimated median (95% CI) CD4 and CD8 cell count (cells/mm^3^) and the CD4:CD8 ratio at 8 years of virologically suppressive ART for an average individual (37-year old male in the heterosexual transmission risk group, born in Western Europe/North America, a CD4 count of 350 or 500 cells/mm^3^ and a CD8 count of 800 cells/mm^3^ and HIV RNA of 4.81 copies/ml at the start and with a random intercept and slopes equal to zero). The results of CMV, HCV and smoking status at the start of ART were obtained in the subgroup of cohorts with data available.

|  | Baseline CD4 count: 350 cells/mm^3^ | | | Baseline CD4 count: 500 cells/mm^3^ | | |
| --- | --- | --- | --- | --- | --- | --- |
|  | CD4 cell count | CD8 cell count | Ratio CD4:CD8 | CD4 cell count | CD8 cell count | Ratio CD4:CD8 |
| Region of origin |  |  |  |  |  |  |
| Europe/North America | 784 (770, 797) | 649 (621, 678) | 1.18 (1.14, 1.22) | 910 (892, 928) | 637 (603, 674) | 1.41 (1.35, 1.48) |
| Caribbean/South America | 791 (764, 818) | 636 (595, 680) | 1.20 (1.13, 1.26) | 918 (870, 967) | 625 (579, 674) | 1.44 (1.36, 1.52) |
| Sub Saharan Africa | 705 (683, 727) | 587 (555, 623) | 1.18 (1.12, 1.23) | 825 (740, 915) | 577 (539, 617) | 1.42 (1.34, 1.49) |
| Other | 752 (731, 773) | 648 (612, 685) | 1.15 (1.10, 1.20) | 876 (859, 894) | 636 (596, 679) | 1.38 (1.31, 1.46) |
| Transmission risk group |  |  |  |  |  |  |
| IDU | 727 (706, 748) | 671 (632, 713) | 1.02 (0.97, 1.07) | 849 (822, 876) | 660 (616, 708) | 1.23 (1.17, 1.30) |
| MSM | 787 (772, 802) | 658 (633, 683) | 1.16 (1.12, 1.19) | 914 (892, 936) | 647 (614, 680) | 1.39 (1.34, 1.45) |
| Heterosexual | 783 (766, 801) | 649 (621, 678) | 1.18 (1.14, 1.22) | 909 (885, 934) | 637 (603, 674) | 1.48 (1.38, 1.57) |
| Other | 765 (744, 785) | 630 (592, 672) | 1.16 (1.12, 1.21) | 890 (863, 917) | 619 (576, 666) | 1.40 (1.33, 1.47) |
| HIV RNA (log_10_ copies/ml) at the start of ART |  |  |  |  |  |  |
| 4.16 | 747 (733, 762) | 643 (615, 672) | 1.12 (1.08, 1.16) | 871 (850, 893) | 632 (597, 668) | 1.35 (1.29, 1.40) |
| 4.81 | 784 (770, 799) | 649 (621, 678) | 1.18 (1.14, 1.22) | 911 (889, 933) | 637 (603, 674) | 1.41 (1.35, 1.48) |
| 5.18 | 811 (795, 826) | 659 (630, 689) | 1.22 (1.18, 1.26) | 939 (917, 962) | 647 (612, 684) | 1.46 (1.40, 1.53) |
| CMV status at the start of ART |  |  |  |  |  |  |
| CMV- | 735 (695, 776) | 496 (450, 546) | 1.42 (1.32, 1.53) | 819 (763, 876) | 476 (421, 538) | 1.64 (1.49, 1.79) |
| CMV+ | 719 (691, 748) | 607 (558, 659) | 1.16 (1.09, 1.24) | 802 (755, 850) | 583 (521, 651) | 1.35 (1.24, 1.47) |
| Smoking status |  |  |  |  |  |  |
| Non-smoker | 733 (712, 755) | 608 (565, 654) | 1.13 (1.07, 1.18) | 858 (823, 893) | 596 (543, 654) | 1.33 (1.24, 1.41) |
| Smoker | 799 (776, 822) | 677 (629, 728) | 1.10 (1.05, 1.16) | 928 (892, 965) | 663 (605, 727) | 1.30 (1.22, 1.38) |
| HCV status at the start of ART |  |  |  |  |  |  |
| HCV- | 779 (758, 799) | 646 (619, 673) | 1.06 (0.98, 1.15) | 905 (871, 939) | 634 (601, 669) | 1.27 (1.15, 1.40) |
| HCV+ | 736 (702, 770) | 658 (622, 697) | 0.99 (0.93, 1.05) | 858 (815, 903) | 646 (605, 691) | 1.18 (1.08, 1.29) |

IDU: intravenous drug use, MSM: men having sex with men, HCV: hepatitis C virus, CMV: cytomegalovirus
