# Supplementary material for: Determinants of Restoration of CD4 and CD8 Cell Counts and Their Ratio in HIV-1–Positive Individuals With Sustained Virological Suppression on Antiretroviral Therapy
Source: J Acquir Immune Defic Syndr. 2018 Dec 3;80(3):292–300. doi: 10.1097/QAI.0000000000001913 (PMC6392208; doi:10.1097/QAI.0000000000001913)
Supplement: SUPPLEMENTARY MATERIAL [file qai-80-292-s002.docx]

Supplementary Table 1 Major PI resistance mutations found in 148/339 patients on GART.

| **Major PI Mutations** | | | n (%) |  |
| --- | --- | --- | --- | --- |
|  |  |  |  |  |
|  |  | 32I | 8 (2) |  |
|  |  | 33F | 29 (9) |  |
|  |  | 46IL | 102 (30) |  |
|  |  | 47VA | 14 (4) |  |
|  |  | 48VM | 9 (3) |  |
|  |  | 50VL | 11(3) |  |
|  |  | 54VTALM | 105 (31) |  |
|  |  | 76V | 42 (12) |  |
|  |  | 82AFTS | 105 (31) |  |
|  |  | 84V | 27 (8) |  |
|  |  | 88S | 0 (0) |  |
|  |  | 90M | 21 (6) |  |
|  |  | |  |  |

Supplementary Table 2 Major reverse transcriptase resistance mutations found in 227/339 patients on GART

| **Mutations** | | | n (%) |  |
| --- | --- | --- | --- | --- |
| TAMS | |  |  |  |
|  |  | 41L | 75 (22) |  |
|  |  | 67N | 106 (31) |  |
|  |  | 70R | 67 (20) |  |
|  |  | 210W | 0 (0) |  |
|  |  | 215FY | 116 (34) |  |
|  |  | 219QE | 0 (0) |  |
| Other NRTI mutations | | |  |  |
|  |  | 65R | 18 (5) |  |
|  |  | 69ins | 0 (0) |  |
|  |  | 70E | 4 (1) |  |
|  |  | 74VI | 20 (6) |  |
|  |  | 115F | 7 (2) |  |
|  |  | 151M | 0 (0) |  |
|  |  | 184VI | 156 (46) |  |
| NNRTI mutations | | |  |  |
|  |  | 100I | 2 (1) |  |
|  |  | 101EP | 27 (8) |  |
|  |  | 103NS | 115 (34) |  |
|  |  | 106AM | 37 (11) |  |
|  |  | 181CIV | 22 (6) |  |
|  |  | 188LCH | 26 (8) |  |
|  |  | 190ASE | 46 (14) |  |
|  |  | 230L | 4 (1) |  |

Supplementary Table 3. Protease inhibitor resistance in 339 patients (Scored using Stanford Version 6.3.1)

| Category | Lopinavir | Atazanavir | Darunavir |
| --- | --- | --- | --- |
|  |  |  |  |
| No resistance mutations | 20 | 20 | 20 |
| Susceptible | 175 | 175 | 241 |
| Potential low-level resistance | 5 | 2 | 11 |
| Low-level resistance | 10 | 16 | 52 |
| Intermediate resistance | 53 | 81 | 13 |
| High-level resistance | 76 | 45 | 2 |
|  |  |  |  |
